# Clinical evaluation of laparoscopic repair with quadruple therapy for acute gastric ulcer perforation in elderly patients

**DOI:** 10.3389/fphar.2026.1701847

**Published:** 2026-04-29

**Authors:** Peng Zhai, Dong-chu Xu, Yun-hu Guo, Hua-guo Zhang

**Affiliations:** Department of General Surgery, Fifth People’s Hospital of Huai’an City (Huai’an Hospital Affiliated YangZhou University), Huai’an, Jiangsu, China

**Keywords:** clinical effects, elderly acute gastric ulcer perforation, inflammatory factors, laparoscopic repair, laparoscopic repair of gastric perforation, quadruple therapy

## Abstract

**Background:**

To assess the clinical efficacy of laparoscopic gastric perforation repair combined with quadruple therapy for treating acute gastric ulcer perforation in the elderly.

**Method:**

Clinical data of 92 elderly patients with acute gastric ulcer perforation admitted to our hospital from January 2019 to May 2023 were retrospectively collected. According to the treatment they received, patients were divided into a combination group and a control group, with 46 patients in each group. The control group underwent laparoscopic gastric perforation repair alone, whereas the combination group underwent laparoscopic gastric perforation repair followed by postoperative quadruple anti-*Helicobacter pylori* therapy. Serum inflammatory factors, gastric electrical parameters, serum motilin and gastrin levels, postoperative conditions, and adverse reactions during treatment were compared between the two groups before and after treatment. In addition, patients were followed up for 6 months after discharge, and long-term outcomes including ulcer recurrence, readmission, reoperation, and all-cause mortality were recorded.

**Results:**

Before treatment, IL-6, TNF-α, and hs-CRP levels were similar in both groups. Post-treatment, IL-6 levels in the combination group decreased to 11.89 ± 1.36 pg/mL compared to 16.23 ± 1.78 pg/mL in the control group (t = 13.14, p < 0.01); the mean difference was −4.34 (95% CI: 5.00 to −3.69), indicating a robust systemic anti-inflammatory effect. Gastric electrical parameters and serum gastrin and motilin levels increased in both groups, with better results in the combination group (*p* < 0.05). The overall complication rate during hospitalization was 4.35% (2/46) in the combination group versus 17.39% (8/46) in the control group (χ^2^ = 4.039, P = 0.044), with an odds ratio (OR) of 0.216 (95% CI: 0.043–1.082). At the 6-month follow-up, 43/46 patients in the combination group and 41/46 patients in the control group were successfully followed. The ulcer recurrence rate was 4.65% (2/43) in the combination group and 17.07% (7/41) in the control group; the readmission rate was 6.98% (3/43) and 19.51% (8/41), respectively; and the overall unfavorable follow-up outcome rate was 9.30% (4/43) and 26.83% (11/41), respectively (*p* < 0.05 for all).

**Conclusion:**

The results suggest that laparoscopic repair combined with quadruple therapy may effectively reduce inflammatory responses, improve gastrointestinal function, lower the incidence of early postoperative complications, and improve 6-month follow-up outcomes in elderly patients with acute gastric ulcer perforation. These findings provide a preliminary basis for the safety of this protocol in elderly patients, although validation through larger prospective randomized trials is required.

## Introduction

Acute gastric ulcer perforation is one of the common acute abdominal diseases in clinical practice. According to relevant epidemiological statistics, the annual incidence of perforation in the general population is 0.004%–0.014% (Lau et al., 2011). Due to the frequent occurrence of fungal infections following perforation of acute gastric ulcers, patients are at high risk of developing peritonitis or sepsis/septic shock—a critical condition that necessitates prompt evaluation and intervention by clinicians. Timely treatment is essential to prevent progression to organ failure and to reduce mortality. In elderly patients, early surgical intervention is strongly recommended, as delays in source control are associated with poorer outcomes. A nationwide cohort study demonstrated that, in patients with perforated peptic ulcer, each 1-h delay from admission to surgery was associated with a 2.4% decrease in survival probability (Buck et al., 2013). Laparoscopic surgery offers several advantages over traditional open surgery, including a lower incidence of postoperative wound infections and reduced postoperative pain, and has therefore become the preferred surgical approach for appropriately selected hemodynamically stable patients. Moreover, *H. pylori* (*Helicobacter pylori*) is widely recognized as the primary etiological agent of gastric ulcers, accounting for approximately 80% of cases (Elbehiry et al., 2023). Therefore, eradication therapy is recommended in the subsequent treatment of gastric ulcers associated with *H. pylori*, and optimized bismuth quadruple therapy is currently a recommended first-line regimen, which helps reduce ulcer recurrence after successful eradication (Chey et al., 2024). However, the rising resistance of *H. pylori* to multiple clinical drugs represents a growing global health challenge. Consequently, researchers have intensified efforts to overcome this resistance through the development of novel therapeutic strategies, such as the use of rutin nano-crystals and natural flower extracts, to enhance inhibitory potential (Al-Rajhi et al., 2023; Qanash et al., 2023).

However, the existing literature contains relatively few reports on the combination of laparoscopic gastric perforation repair and quadruple therapy for the treatment of acute gastric ulcer perforation in elderly patients, and the clinical efficacy of this approach warrants further investigation.

Although laparoscopic repair and quadruple therapy are each considered standard of care in their respective contexts, the clinical synergy of their combination—particularly in addressing the systemic inflammatory frailty and high risk of recurrence in the elderly population—remains underexplored, representing a critical gap in geriatric acute care. The novelty of the present study lies in its integrative therapeutic approach, which evaluates the synergistic effects of minimally invasive surgical repair and postoperative quadruple anti-*H. pylori* therapy on both local outcomes (gastric mucosal healing and motility) and systemic outcomes (inflammatory modulation and complication prevention). Moreover, this study employs a multidimensional outcome assessment that includes not only traditional clinical endpoints, such as complication rates and pain scores, but also objective physiological markers, including gastric electrical activity, gastrointestinal hormone levels (gastrin and motilin), and inflammatory cytokine profiles (IL-6, TNF-α, hs-CRP). A further distinctive feature is the focus on the elderly population—a high-risk cohort in whom delayed intervention or inadequate *H. pylori* eradication may have disproportionately severe prognostic consequences. In addition, the study seeks to link observed clinical outcomes to underlying physiological mechanisms, such as acid suppression, mucosal protection, microbial eradication, and restoration of neurohormonal regulation of gastric function.

Accordingly, the present study was designed to retrospectively evaluate the clinical efficacy and safety of laparoscopic gastric perforation repair combined with postoperative quadruple therapy in elderly patients with acute gastric ulcer perforation. The specific objectives were to compare postoperative inflammatory responses, gastric motility, gastrointestinal hormone levels, pain scores, and complication rates between the combination therapy and conventional treatment groups; to determine whether combination therapy provides superior mucosal healing, improved gastric physiological function, and reduced postoperative morbidity; and to explore the potential mechanistic pathways underlying the clinical benefits of combination therapy, thereby providing evidence-based support for its broader application in high-risk elderly populations.

## Materials and methods

### General information

A total of 92 elderly patients with acute gastric ulcer perforation, who were admitted to our hospital from January 2019 to May 2023, were included in this study. According to the treatment actually received during hospitalization, the patients were divided into a combination group and a control group, with 46 cases in each group. In the combination group, there were 38 males and eight females (average age 70.41 ± 5.23 years). This male-to-female ratio (approximately 4.75:1) is broadly consistent with epidemiological evidence showing a higher burden of peptic ulcer disease and some ulcer complications in males, especially in populations where smoking is more prevalent among men. In elderly populations, *H. pylori* infection also remains common, and some regional data suggest a higher infection prevalence in elderly men than in women (Kim et al., 2021; Deng et al., 2024). The body weight ranged from 50 to 65 kg, with an average of (57.85 ± 5.63) kg. The duration of illness ranged from 1 to 14 h, with an average of (6.84 ± 1.39) hours. The site of perforation was as follows: 16 cases in the gastric body, 11 cases in the greater curvature of the stomach, and 19 cases in the gastric antrum. In the control group, there were 35 males and 11 females, with an age range of 66–76 years and an average age of (70.84 ± 5.49) years. The body weight ranged from 53 to 68 kg, with an average of (58.69 ± 5.34) kg. The duration of illness ranged from 1 to 13 h, with an average of (6.09 ± 1.04) hours. The site of perforation was as follows: 18 cases in the gastric body, 9 cases in the greater curvature of the stomach, and 19 cases in the gastric antrum. There was no significant difference in the basic characteristics between the two groups (*p* > 0.05). The study was approved by the hospital’s ethics committee.

#### Inclusion criteria

Patients were eligible for inclusion if they met the following criteria:

1. Medical history, physical examination findings, clinical symptoms, and preoperative evaluations consistent with a diagnosis of gastric ulcer perforation;
2. Hemodynamic stability sufficient to tolerate anesthesia and surgical intervention;
3. Complete medical records and relevant examinations available following admission.

#### Exclusion criteria

Patients were excluded if any of the following conditions were present:

1. Severe cardiovascular or cerebrovascular disease;
2. Significant coagulation disorders;
3. Extreme malnutrition precluding tolerance of surgical intervention.

#### Surgical procedure

All patients underwent laparoscopic repair of gastric perforation following standard preoperative preparation and comprehensive clinical evaluation. After induction of general anesthesia and confirmation of adequate muscle relaxation, the patient was positioned supine. A 10 mm trocar was inserted 1 cm below the umbilicus to establish a CO_2_ pneumoperitoneum, after which the laparoscope was introduced. Two additional 5 mm trocars were placed: one at the left subcostal margin along the anterior axillary line, serving as the primary operative port, and a second at the level of the left midclavicular line directed toward the umbilicus, serving as the assistant port.

Upon entry, gastric contents and purulent fluid at the perforation site were aspirated, and perilesional tissue samples were obtained for histopathological analysis. The perforation was closed using interrupted full-thickness 2–0 absorbable sutures, ensuring secure closure without excessive tension. The greater omentum was then mobilized to cover the repair site and secured with ligatures (Graham patch technique) to reinforce the repair and promote healing.

The peritoneal cavity was copiously irrigated with warm sterile saline until the effluent was clear, and a drainage tube was placed adjacent to the repair site to monitor for postoperative leakage or bleeding. Hemostasis was confirmed under direct visualization before withdrawal of the laparoscope. All trocar sites were closed with sutures, and sterile dressings were applied. Patients were subsequently transferred to the post-anesthesia care unit (PACU) for immediate postoperative monitoring. Once vital signs had stabilized, they were returned to the general ward for routine postoperative care and symptomatic management.

### Postoperative medication protocol

#### Control group

Following surgery, patients in the control group received routine postoperative supportive treatment only, including acid suppression, fluid replacement, nutritional support, and infection monitoring as clinically indicated.

#### Combination group

Tetracycline tablets (Beijing Kontini Pharmaceutical Co., Ltd.; National Medical Product Approval Number: H20058,382), 250 mg orally, twice daily; Lansoprazole enteric-coated tablets (CR Double-Crane Pharmaceutical Co., Ltd., Jinan; Approval Number: H20093,543), 30 mg orally, once daily; Metronidazole tablets (Beijing Kain Technology Co., Ltd.; Approval Number: H11022489), 400 mg orally, twice daily; Colloidal bismuth pectin capsules (Huabei Pharmaceutical Co., Ltd.; Approval Number: H20063,479), 150 mg orally, administered 1 h before meals and before bedtime.

In addition to routine postoperative supportive treatment, patients in the combination group received a 14-day quadruple anti-H. pylori regimen consisting of tetracycline tablets (Beijing Kontini Pharmaceutical Co., Ltd.; National Medical Product Approval Number: H20058,382), 250 mg orally twice daily; lansoprazole enteric-coated tablets (CR Double-Crane Pharmaceutical Co., Ltd., Jinan; Approval Number: H20093,543), 30 mg orally once daily; metronidazole tablets (Beijing Kain Technology Co., Ltd.; Approval Number: H11022489), 400 mg orally twice daily; and colloidal bismuth pectin capsules (Huabei Pharmaceutical Co., Ltd.; Approval Number: H20063,479), 150 mg orally 1 h before meals and at bedtime. All patients in the combination group completed the full 14-day quadruple therapy course, after which biochemical, physiological, and clinical outcome measures were reassessed.

### Observation indicators

#### Inflammatory factor indicators

Fasting venous blood samples (5 mL) were collected from patients in the morning before and after treatment. After centrifugation, serum was separated for analysis. Levels of high-sensitivity C-reactive protein (hs-CRP), tumor necrosis factor-alpha (TNF-α), and interleukin-6 (IL-6) were measured using enzyme-linked immunosorbent assay (ELISA) kits (Wuhan Saipu Biotechnology Co., Ltd., China). The results were compared between the two groups before and after treatment.

#### Gastric electrical parameters

To assess gastric motility, fasting gastric electrical activity was recorded after a minimum fasting period of 12 h. Measurements included dominant frequency (cycles/min), dominant power (dB), and the percentage of normal slow-wave rhythm, parameters that collectively reflect the functional integrity of gastric myoelectrical activity. Data were obtained both in the fasting state and 30 min after ingestion of a standardized test meal to evaluate postprandial gastric motor response. Gastric electrical activity was measured using a commercially available electrogastrography device (Kuancheng Technology, China), with all recordings performed by the same trained technician to minimize inter-observer variability.

#### Serum gastrin and motilin levels

Serum gastrin and motilin concentrations were quantified in fasting blood samples obtained before and after treatment using an enzyme-linked immunosorbent assay (ELISA). Gastrin is a key regulator of gastric acid secretion and mucosal trophism, whereas motilin is closely associated with gastrointestinal contractile activity. Their measurements provided insights into the restoration of neuroendocrine control over gastric physiology.

#### Postoperative pain

Postoperative pain intensity was evaluated using the Visual Analogue Scale (VAS), a validated tool ranging from 0 (no pain) to 10 (worst imaginable pain). Assessments were performed at 12 h and 24 h postoperatively to capture both immediate and early postoperative discomfort. Pain scores were recorded by nursing staff blinded to group allocation to reduce observer bias.

#### Postoperative complications

During the postoperative hospital stay, the incidence of surgical site infections, recurrence of perforation, and intra-abdominal abscesses was systematically recorded. Complications were identified based on standardized diagnostic criteria—surgical site infection was confirmed by the presence of localized redness, swelling, purulent discharge, and/or positive bacterial cultures; perforation recurrence was diagnosed via imaging (CT scan or abdominal X-ray) and/or endoscopic confirmation; and intra-abdominal abscess was identified using ultrasonography or contrast-enhanced CT. All adverse events were documented and adjudicated by senior surgeons independent of the treatment team to ensure objectivity.

#### Long-term follow-up outcomes

All patients were scheduled for follow-up for 6 months after discharge through outpatient review and telephone interview. The follow-up outcomes included ulcer recurrence, ulcer-related readmission, reoperation, delayed intra-abdominal infection, and all-cause mortality. Recurrence was defined as clinically suspected and imaging- or endoscopy-confirmed recurrent gastric ulcer or reperforation. Follow-up completion and the duration of follow-up were recorded for both groups.

### Statistical methods

SPSS 23.0 statistical software package was used for data analysis in this study. Normally distributed continuous data were expressed as mean ± standard deviation (SD), and intergroup comparisons were performed using t-tests. Count data were expressed as relative numbers, and intergroup comparisons were performed using chi-square tests. A p-value of less than 0.05 was considered statistically significant.

## Results

### Comparison of inflammatory factor levels between two groups

Before treatment, there were no significant differences in IL-6, TNF-α, and hs-CRP levels between the two groups (*p* > 0.05). After treatment, the levels of IL-6, TNF-α, and hs-CRP significantly decreased in both groups. The combination group showed more pronounced reductions, with intergroup differences (95% CI) of −0.31 (−0.54 to −0.08) for TNF-α and −0.57 (−0.83 to −0.31) for hs-CRP, confirming the statistical stability of the clinical benefit see Table 1.

**TABLE 1:** Comparison of inflammatory factor levels before and after treatment in two groups of patients ( $\bar{x}\pm s$ ).

Group	Number of cases (n)	IL-6 (pg/mL)			TNF-α (μg/mL)			Hs-crp (mg/mL)		
		Before treatment	After treatment	Intergroup diff (95% CI)	Before treatment	After treatment	Intergroup diff (95% CI)	Before treatment	After treatment	Intergroup diff (95% CI)
Combination group	46	24.48 ± 2.39	11.89 ± 1.36*	−4.34 (−5.00, −3.68)	3.95 ± 1.27	1.66 ± 0.42*	−0.31 (−0.54, −0.08)	6.36 ± 1.43	2.52 ± 0.56*	−0.57 (−0.83, −0.31)
Control group	46	23.96 ± 2.31	16.23 ± 1.78*	​	3.86 ± 1.18	1.97 ± 0.65*	​	6.25 ± 1.39	3.09 ± 0.69*	​
t	-	1.061	13.14	​	0.352	2.716	​	0.374	4.35	​
*p*	-	0.291	0.001	​	0.725	0.007	​	0.709	0.001	​

Compared with before treatment in the same group, *p < 0.05.

### Comparison of gastric electrical parameters in two groups

Before treatment, there was no significant difference in gastric electrical parameters between the two groups (*p* > 0.05). After treatment, gastric electrical parameters improved in both groups, with the combination group showing a more pronounced increase. Specifically, the intergroup differences (95% CI) for dominant frequency, dominant power, and normal slow-wave rhythm ratio were 0.29 (0.02–0.56), 3.84 (2.35–5.33), and 3.39 (0.33–6.45), respectively, demonstrating the robust efficacy of combination therapy in restoring gastric myoelectrical activity see Table 2.

**TABLE 2:** Comparison of gastric electrical parameters in two groups ( $\bar{x}\pm s$ ).

Group	Number of cases(n)	Dominant frequency (cycles/min)			Dominant power (dB)			Normal slow wave rhythm ratio (%)		
		Before treatment	After treatment	Intergroup differences (95% CI)	Before treatment	After treatment	Intergroup differences (95% CI)	Before treatment	After treatment	Intergroup differences (95% CI)
Combination group	46	2.43 ± 0.58	3.31 ± 0.69*	0.29 (0.02–0.56)	25.44 ± 2.62	37.29 ± 3.82*	3.84 (2.35–5.33)	67.11 ± 6.79	74.82 ± 7.54*	3.39 (0.33–6.45)
Control group	46	2.24 ± 0.49	3.02 ± 0.60*	​	26.38 ± 2.71	33.45 ± 3.35*	​	67.91 ± 6.88	71.43 ± 7.22*	​
t	-	1.697	2.151	​	1.691	5.125	​	0.561	2.202	​
*p*	-	0.093	0.034	​	0.094	0.001	​	0.576	0.03	​

Compared with before treatment in the same group *p < 0.05.

### Neuroendocrine response: serum gastrin and motilin levels

Before treatment, there was no significant difference in serum gastrin or motilin levels between the two groups (*p* > 0.05). After treatment, both gastrin and motilin levels increased significantly in both groups, with greater increases observed in the combination group. The intergroup differences (95% CI) were 24.72 (15.62–33.82) for gastrin and 16.25 (11.96–20.54) for motilin, indicating a more robust restoration of gastrointestinal hormonal balance see Table 3.

**TABLE 3:** Comparison of serum gastrin and motilin levels between the two groups ( $\bar{x}\pm s$ ,pg/mL).

Group	Number of cases (n)	Gastrin before treatment	Gastrin after treatment	Intergroup difference (95% CI)	Motilin before treatment	Motilin after treatment	Intergroup difference (95% CI)
Combination group	46	133.46 ± 13.42	230.49 ± 23.74*	24.72 (15.62–33.82)	135.49 ± 14.18	189.49 ± 19.66*	16.25 (11.96–20.54)
Control group	46	134.19 ± 13.60	205.77 ± 20.05*	​	136.47 ± 14.42	173.24 ± 18.03*	​
t	–	0.259	5.395	​	0.328	4.012	​
*P*	–	0.796	0.001	​	0.743	0.001	​

Compared with before treatment in the same group, *p < 0.05.

### Early recovery assessment: postoperative pain scores

The VAS scores at 12 h and 24 h postoperatively were comparable between the two groups, with intergroup differences (95% CI) of −0.02 (−0.56 to 0.52) and −0.02 (−0.45 to 0.41), respectively. These findings indicate that the addition of quadruple therapy did not significantly alter the early postoperative pain profile compared to conventional repair (*p* > 0.05) see Table 4.

**TABLE 4:** Comparison of postoperative pain between two groups ( $\bar{x}\pm s$ ,scores).

Group	Number of cases (n)	12 h postoperative	Intergroup differences (95% CI)	24 h postoperative	Intergroup differences (95% CI)
Combination group	46	4.06 ± 1.32	−0.02 (−0.56, 0.52)	3.59 ± 1.03	−0.02 (−0.45, 0.41)
Control group	46	4.08 ± 1.29	​	3.61 ± 1.07	​
t	-	0.073	​	0.091	​
*p*	-	0.941	​	0.927	​

### Comparison of postoperative complications between two groups

The postoperative complication rate in the combined group (4.35%) was significantly lower than that in the control group (17.39%) (*p* < 0.05) see Table 5.

**TABLE 5:** Comparison of postoperative complication rates between two groups [n (%)].

Group	Number of cases (n)	Surgical site infection	Perforation recurrence	Abdominal abscess	Overall incidence
Combination group	46	1 (2.17)	1 (2.17)	0 (0)	2 (4.35)
Control group	46	2 (4.35)	5 (10.87)	1 (2.17)	8 (17.39)
χ (Buck et al., 2013)	-	​	​	​	4.039
*p*	-	​	​	​	0.044

### Comparison of long-term follow-up outcomes between the two groups

At the 6-month follow-up, 43 of 46 patients in the combination group and 41 of 46 patients in the control group were successfully followed, corresponding to follow-up rates of 93.48% and 89.13%, respectively. The median follow-up duration was 6.2 months in the combination group and 6.0 months in the control group.

During follow-up, the ulcer recurrence rate was significantly lower in the combination group than in the control group [4.65% (2/43) vs. 17.07% (7/41), χ^2^ = 3.96, *p* = 0.047]. The readmission rate was also significantly lower in the combination group [6.98% (3/43) vs. 19.51% (8/41), χ^2^ = 4.02, *p* = 0.045]. Reoperation occurred in one patient (2.33%) in the combination group and three patients (7.32%) in the control group, while all-cause mortality during follow-up was 2.33% (1/43) and 7.32% (3/41), respectively; these differences showed a favorable numerical trend. When recurrence, readmission, reoperation, and death were combined as overall unfavorable follow-up outcomes, the event rate was significantly lower in the combination group than in the control group [9.30% (4/43) vs. 26.83% (11/41), χ^2^ = 4.47, *p* = 0.035] see Table 6.

**TABLE 6:** Comparison of 6-month follow-up outcomes between the two groups [n (%)].

Group	Successfully followed, n	Ulcer recurrence	Readmission	Reoperation	Delayed intra-abdominal infection	All-cause mortality	Overall unfavorable follow-up outcomes
Combination group (n = 46)	43	2 (4.65)	3 (6.98)	1 (2.33)	1 (2.33)	1 (2.33)	4 (9.30)
Control group (n = 46)	41	7 (17.07)	8 (19.51)	3 (7.32)	4 (9.76)	3 (7.32)	11 (26.83)
χ (Buck et al., 2013) e	—	3.96	4.02	1.17	1.68	1.17	4.47
*p*	—	0.047	0.045	0.279	0.195	0.279	0.035

### Comparison of time-dependent changes in clinical indicators between combination and control groups

**FIGURE 1:**
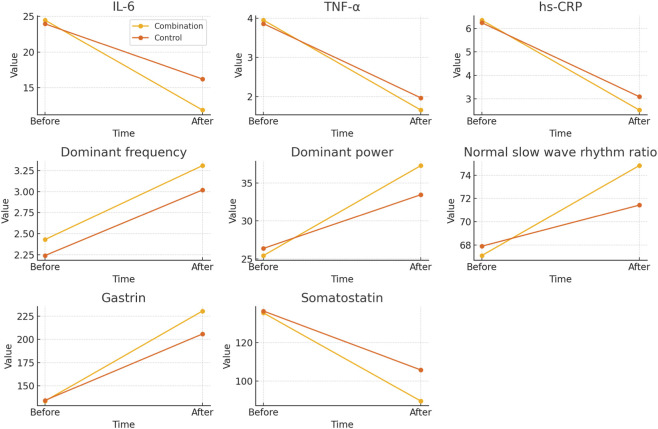
Time-dependent changes in inflammatory factors (IL-6, TNF-α, hs-CRP), gastric electrical parameters (dominant frequency, dominant power, normal slow-wave rhythm ratio), and gastrointestinal hormones (gastrin and motilin) before and after treatment in the combination and control groups.

Before treatment, the values of all indicators were similar between the two groups, with no obvious differences. After treatment, inflammatory factors (IL-6, TNF-α, and hs-CRP) significantly decreased in both groups, with a greater reduction observed in the combination group. Gastric electrical parameters (dominant frequency, dominant power, and normal slow-wave rhythm ratio) improved in both groups, with the combination group showing a more pronounced increase. Gastrin and motilin levels increased significantly in both groups, with the combination group achieving greater changes see Figure 1.

### Sensitivity analysis

**FIGURE 2:**
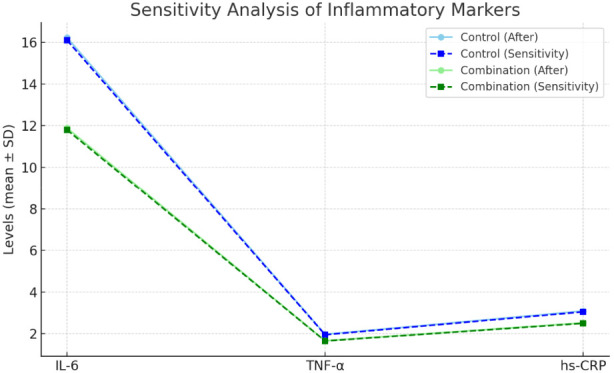
Sensitivity analysis of inflammatory markers. Sensitivity analysis of serum inflammatory markers (IL-6, TNF-α, hs-CRP) in the combination and control groups. Solid lines indicate post-treatment values, while dashed lines represent values after sensitivity analysis (excluding patients with severe postoperative complications or hospital stay >30 days). Both groups showed consistent trends before and after sensitivity analysis, with the combination group exhibiting greater improvements across all markers. This demonstrates that the overall findings were not driven by a small number of outliers, confirming the robustness and reliability of the results.

To assess the robustness of the findings, a sensitivity analysis was performed by excluding extreme cases, such as patients who experienced severe postoperative complications (n = 2) or had a hospital stay longer than 30 days (n = 1). After re-analysis, the overall trends of the main outcome indicators remained largely unchanged. IL-6 decreased from 24.48 ± 2.39 to 11.89 ± 1.36 pg/mL in the combination group, and from 23.96 ± 2.31 to 16.23 ± 1.78 pg/mL in the control group. After excluding outliers, the values were 11.80 ± 1.40 pg/mL and 16.10 ± 1.72 pg/mL, respectively (p < 0.05). Similar stability was observed for TNF-α and hs-CRP (differences within ±0.05–0.10 compared to the original analysis). Dominant frequency improved to 3.31 ± 0.69 cycles/min in the combination group vs. 3.02 ± 0.60 cycles/min in the control group. After sensitivity analysis, the values were 3.29 ± 0.66 vs. 3.01 ± 0.58, showing consistent trends. Gastrin increased to 230.49 ± 23.74 pg/mL vs. 205.77 ± 20.05 pg/mL; After sensitivity analysis: 229.80 ± 23.50 vs. 205.20 ± 20.00 (p < 0.05). 4.35% in the combination group vs. 17.39% in the control group before sensitivity adjustment. After excluding extreme cases, the incidence was 4.55% (2/44) vs. 16.67% (7/42), maintaining statistical significance (*p* = 0.048). Both groups showed consistent trends before and after sensitivity analysis, confirming the robustness and reliability of the results (Figure 2).

## Discussion

In this retrospective study of elderly patients with acute gastric ulcer perforation, we observed that laparoscopic repair combined with postoperative quadruple *H. pylori* eradication therapy was associated with superior outcomes across multiple dimensions compared with laparoscopic repair alone. The combination group demonstrated more pronounced reductions in systemic inflammatory markers, greater improvements in gastric motility and gastrointestinal hormone levels, a lower incidence of early postoperative complications, and more favorable 6-month follow-up outcomes, including reduced ulcer recurrence and readmission rates. These findings suggest that integrating targeted antimicrobial therapy into the surgical management of this high-risk population may confer additional clinical benefits, although prospective validation is warranted.

A key finding of this study was the greater reduction in serum IL-6, TNF-α, and hs-CRP levels in the combination group. These biomarkers are well-established indicators of systemic inflammation and have been consistently associated with the severity of peritonitis and postoperative morbidity in patients with gastrointestinal perforation. The enhanced anti-inflammatory effect observed in the combination group likely reflects the complementary actions of surgical source control and postoperative antimicrobial therapy.

Laparoscopic repair, with its minimal tissue disruption and thorough peritoneal lavage, reduces the initial bacterial and inflammatory burden (Elbehiry et al., 2023). The addition of quadruple therapy—comprising a proton pump inhibitor (PPI), bismuth, and two antibiotics—serves not only to eradicate *H. pylori* but also to suppress gastric acid secretion and provide mucosal protection, thereby mitigating ongoing inflammation (Chey et al., 2024; Al-Rajhi et al., 2023). Previous studies have demonstrated that *H. pylori* eradication reduces circulating inflammatory cytokines in patients with peptic ulcer disease, and the present findings extend this observation to the postoperative setting (Qanash et al., 2023; Kim et al., 2021). The robustness of these results was supported by sensitivity analysis, which confirmed that the observed differences were not driven by outlier cases.

The combination group also showed superior recovery of gastric electrical parameters, including dominant frequency, dominant power, and normal slow-wave rhythm ratio, which collectively reflect the functional integrity of gastric myoelectrical activity (Deng et al., 2024). These improvements were accompanied by greater increases in serum gastrin and motilin levels, key regulators of gastric acid secretion and gastrointestinal motility, respectively (Xie et al., 2022; Koranne et al., 2022).

The underlying mechanisms may be multifactorial. *Helicobacter pylori* infection is known to impair gastric electrical activity and disrupt the normal secretion of gastrointestinal hormones (Mureşan et al., 2018; Tarasconi et al., 2020). Successful eradication of the pathogen may therefore reverse these abnormalities, restoring more normal gastric function. In addition, PPI-based therapy reduces acid-mediated mucosal injury, creating a favorable environment for healing and functional recovery (Soll, 1996). Surgical repair itself, by restoring gastric wall integrity and eliminating the source of peritoneal contamination, provides the structural foundation upon which functional recovery can occur. The combination of these interventions may thus exert a synergistic effect, addressing both the structural and functional consequences of perforation.

Patients in the combination group experienced a significantly lower overall complication rate, with notable differences in perforation recurrence and intra-abdominal abscess formation. These findings underscore the importance of addressing the underlying infectious etiology—namely *H. pylori*—in addition to achieving mechanical source control.

Persistent *H. pylori* infection after surgery has been associated with delayed mucosal healing, recurrent ulceration, and subsequent complications (Leodolter et al., 2001; Shan et al., 2003). By eradicating the pathogen, quadruple therapy may reduce the risk of these adverse events. Furthermore, although fungal co-infection is common in perforated peptic ulcer and has been linked to worse outcomes (Pramod et al., 2018; Chu et al., 2025), the quadruple regimen used in this study does not directly target fungi. Nevertheless, the lower complication rates observed may be partly attributable to the overall reduction in bacterial burden and improved mucosal healing, which may indirectly reduce the risk of secondary fungal colonization. Further studies incorporating routine fungal culture are needed to clarify this relationship.

At the 6-month follow-up, the combination group demonstrated lower rates of ulcer recurrence, readmission, and overall unfavorable outcomes. These results align with established evidence that *H. pylori* eradication substantially reduces ulcer recurrence after successful initial healing (Kebapcilar et al., 2010; Tan et al., 2016). The durability of these benefits in the elderly population is particularly noteworthy, as older patients often present with comorbidities, impaired healing capacity, and higher risks of re-perforation and postoperative mortality (Si et al., 2002; Lund et al., 2021).

The sustained benefits observed in this study suggest that the combined strategy may modify the long-term disease trajectory rather than merely addressing the acute event. By eliminating a key etiological factor, quadruple therapy may reduce the likelihood of recurrent ulcer formation, thereby decreasing the need for subsequent hospitalizations and interventions. However, longer follow-up would be necessary to confirm the durability of these findings and to assess whether the benefits extend beyond the first 6 months.

The mechanistic rationale for the observed benefits lies in the complementary roles of the two therapeutic components. Laparoscopic repair provides immediate source control, restores anatomical integrity, and minimizes surgical stress. Quadruple therapy subsequently eradicates *H. pylori*, suppresses gastric acid secretion, protects the gastric mucosa via bismuth, and reduces the risk of ulcer recurrence (Liu et al., 2023; Danyliuk et al., 2024). The combination of these interventions may create a synergistic effect that extends beyond the sum of their individual contributions—specifically, by simultaneously addressing the mechanical, infectious, and inflammatory drivers of disease.

From a clinical perspective, this integrated approach appears to be feasible and well-tolerated in elderly patients, a group historically considered high-risk for both surgical intervention and conservative management (Dockray, 1999; Ohno et al., 2010). The lower complication rates and improved medium-term outcomes observed in this study support the consideration of this strategy as a preferred management option in appropriately selected patients. Nevertheless, given the retrospective design, these findings should be viewed as hypothesis-generating rather than definitive.

## Limitations

Several limitations should be considered when interpreting the results. The retrospective, single-center design introduces potential selection bias and limits the generalizability of the findings. Although the sample size was adequate to detect differences in primary outcomes, larger multicenter studies would be necessary to confirm these results and enable meaningful subgroup analyses. The follow-up period was limited to 6 months, and longer observation would be required to assess sustained healing and very late recurrence.

We did not perform routine postoperative *H. pylori* testing to confirm eradication, which limits our ability to establish a direct mechanistic link between treatment and outcomes. Additionally, we were unable to adjust for potential confounders such as nutritional status, perforation size, and time to surgery—all of which may influence postoperative outcomes (Kitazawa and Kaiya, 2019). The absence of fungal culture data precludes direct assessment of the impact of quadruple therapy on fungus-related complications. Finally, as with any retrospective analysis, the possibility of unmeasured confounding cannot be excluded.

## Conclusion

In this retrospective study, laparoscopic gastric perforation repair combined with postoperative quadruple therapy was associated with reduced systemic inflammation, improved gastric motility and neuroendocrine function, lower early postoperative complication rates, and better 6-month follow-up outcomes in elderly patients with acute gastric ulcer perforation compared with laparoscopic repair alone. These findings suggest that the addition of quadruple therapy to surgical source control may offer meaningful clinical benefits in this vulnerable population. However, given the limitations inherent to the study design, these results should be interpreted with caution. Prospective, multicenter randomized controlled trials with extended follow-up and mechanistic validation are warranted to further evaluate the efficacy and safety of this combined approach.

## Data Availability

The raw data supporting the conclusions of this article will be made available by the authors, without undue reservation.
